# Phylogeny and systematics of the genus *Clonostachys*

**DOI:** 10.3389/fmicb.2023.1117753

**Published:** 2023-03-03

**Authors:** Yao Wang, De-Xiang Tang, Run Luo, Yuan-Bing Wang, Chinnapan Thanarut, Van-Minh Dao, Hong Yu

**Affiliations:** ^1^Yunnan Herbal Laboratory, College of Ecology and Environmental Sciences, Yunnan University, Kunming, China; ^2^The International Joint Research Center for Sustainable Utilization of Cordyceps Bioresources in China and Southeast Asia, Yunnan University, Kunming, China; ^3^Faculty of Agricultural Production, Maejo University, Chiang Mai, Thailand; ^4^Institute of Regional Research and Development, Ministry of Science and Technology, Hanoi, Vietnam

**Keywords:** Bionectriaceae, molecular systematics, multi-gene phylogeny, morphology, new species

## Abstract

**Introduction:**

*Clonostachys*, a genus with rich morphological and ecological diversity in Bionectriaceae, has a wide distribution among diverse habitats.

**Methods and Results:**

In the present study, a phylogenetic framework is reconstructed for the family Bionectriaceae focusing on *Clonostachys* through increased taxon-sampling using the nr*LSU* sequence. Through surveying *Clonostachys* in China, Vietnam, and Thailand over the past 3 years, seven *Clonostachys* spp. were found and identified. Two new species, *C. chuyangsinensis* and *C. kunmingensis,* are described and illustrated based on morphological characteristics and molecular data. The phylogenetic positions of the seven species were evaluated based on four genomic loci (ITS, nr*LSU*, *TUB2*, and *TEF1*).

**Discussion:**

Moreover, the genetic divergence comparisons of *Clonostachys* species for three markers (ITS, *TUB2*, and *TEF1*) are also provided. The results indicated that the *TEF1* sequence data provided the best resolution for distinguishing species of *Clonostachys*, followed by sequence data for the *TUB2* and ITS regions.

## Introduction

The asexual morph-typified genus *Clonostachys* was established by Corda (1839) on the basis of the type species, *C. araucaria*, which possessed penicillate conidiophores and imbricate conidia held in columns. This species is now considered a synonym of *C. rosea* (Link) Schroers et al. (basionym *Penicillium roseum* Link) (Schroers et al., 1999). *Clonostachys* (Bionectriaceae, Hypocreales) is characterized by penicillate, sporodochial, or dimorphic conidiophores and phialidic conidiogenous cells producing hyaline conidia (Schroers, 2001). Teleomorph, is originially described *Bionectria* (Spegazzini, 1919), and characterized by ascomata typically seated on a pseudoparenchymatous stroma or arising directly on the substrate, being white, pale yellow, or orange to dark brownish-orange, not changing color in 3% KOH or lactic acid, not collapsing or laterally pinched when dry; warted or smooth; an ascomatal wall composed of 1–3 regions with the outer region composed of subglobose to globose, thick-walled cells; ascospores smooth; spinulose, striate or warted (Schroers, 2001; Lechat and Fournier, 2018). Based on the monograph of *Bionectria* and *Clonostachys* by Schroers (2001), this connection was confirmed by DNA sequences. Because *Clonostachys* was described earlier than *Bionectria*, Rossman et al. (2013) recommended *Clonostachys* as the name of this genus.

It is generally agreed that distinguishing individual species of *Clonostachys* using only morphological characteristics can be difficult (Schroers et al., 1999; Abreu et al., 2014). The members of the genus *Clonostachys* were accommodated in *Acrostalagmus*, *Clonostachyopsis*, *Dendrodochium*, *Gliocladium*, *Gliocladochium*, *Myrothecium*, *Sesquicillium*, *Spicaria*, *Verticilliodochium*, or *Verticillium* (Schroers, 2001). It is the huge diversification of morphs of closely related *Clonostachys* species, what did not allow recognition that they all may belong to a single genus, *Clonostachys*. Given the problems with species delimitation in *Clonostachys* using morphology, molecular data are essential to establish robust species boundaries. The first molecular study of *Clonostachys*/*Bionectria* was carried out by Rossman et al. (2001) using large subunit rDNA sequences. The results showed that the genus represents a well-resolved monophyletic lineage. Subsequently, DNA sequences of the internal transcribed spacer regions of the rDNA (ITS rDNA) and a portion of the β-tubulin (*TUB2*) gene were widely used to resolve taxonomic questions for *Clonostachys*/*Bionectria* (Schroers, 2001; Hirooka and Kobayashi, 2007; Luo and Zhuang, 2010; Chen et al., 2016; Prasher and Chauhan, 2017). Regrettably, not all recognized species inside this group formed well-supported clades in these two-gene phylogenies (Moreira et al., 2016). Other DNA sequences recently employed to improve the resolution of phylogenetic trees for the species of *Clonostachys*/*Bionectria* include ATP citrate lyase (*ACL1*), *TUB2*, the large subunit of RNA polymerase II (*RPB1*), and the translation elongation factor 1-α (*TEF1*) gene regions (Moreira et al., 2016). However, sequence data of the above-mentioned four protein-encoding gene regions in GenBank^1^ are incomplete for the group.

There is no doubt that *Clonostachys* belongs to the family Bionectriaceae, but its taxonomic position in relation to other genera is debated within Bionectriaceae (Rossman et al., 2001; Hyde et al., 2020; Schoch et al., 2020). In more recent studies, *Clonostachys* was suggested as a close relative of the genus *Stephanonectria* that was confirmed as a member of Bionectriaceae (Hyde et al., 2020). However, Schoch et al. (2020) reported that *Stephanonectria* was a genus of ascomycetes in the family Nectriaceae (^2^accessed on 1 July 2022). Rossman et al. (2001) found that the genera *Emericellopsis* and *Stanjemonium* belonged to Bionectriaceae in spite of the distant relation to *Clonostachys*, whereas Schoch et al. (2020) placed their taxonomic positions in the Hypocreales genera, *incertae sedis* genera (see Footnote 2 accessed on 1 July 2022). Therefore, it is imperative to reconstruct the phylogenetic framework for the Bionectriaceae focusing on *Clonostachys* through increased taxon sampling.

In the current study, we aimed to: (1) consider the identity of previously unidentified *Clonostachys* isolates collected over a 3-year period from China, Vietnam, and Thailand and (2) re-evaluate the taxonomic stability of *Clonostachys* among related genera within Bionectriaceae and phylogenetic relationships between *Clonostachys* species.

## Materials and methods

### Soil and specimen collection and fungus isolation

Soil samples and fungus-infected spider specimens were collected from 11 locations in 2017 and 2019, including eight different locations within Yunnan Province, China, two locations within Dak Lak Province, Vietnam, and one location in Chiang Mai, Thailand.

*Clonostachys* strains were isolated from the soil samples according to methods described in our previous publication (Wang et al., 2015). Briefly, 2 g of soil was added to a flask containing 20 ml sterilized water and glass beads. The soil suspension was shaken for about 10 min and then diluted 100 times. Subsequently, 200 μL of the diluted soil suspension was spread on Petri dishes with solidified onion garlic agar (OGA: 20 g of grated garlic and 20 g of onion were boiled in 1 l of distilled water for 1 h; the boiled biomass was then filtered-off, and 2% agar was added). Czapek yeast extract agar (CYA, Advanced Technology and Industrial Co., Ltd., China) and potato dextrose agar (PDA, Difco, United States) were used, and all media had 50 mg/L rose Bengal and 100 mg/L kanamycin added. Conidia developing on spider cadavers were transplanted onto plates of PDA and cultured at 25°C. Colonies of the isolated filamentous fungi appearing in the culture were transferred onto fresh PDA media. The purified fungal strain was transferred to PDA slants and cultured at 25°C until its hyphae spread across the entire slope. The emerging fungal spores were washed with sterile physiological saline and made into a spore suspension of 1 × 10^3^ cells/mL. To obtain monospore cultures, a part of the spore suspension was placed on PDA using a sterile micropipette, and then a Petri dish was incubated at 25°C. Specimens and type material were deposited in the Yunnan Herbal Herbarium (YHH) at the Institute of Herb Biotic Resources of Yunnan University, China. Cultures were stored in the Yunnan Fungal Culture Collection (YFCC) at the Institute of Herb Biotic Resources of Yunnan University.

### Morphological observations

Macroscopic characters were collected from colonies grown on PDA and corn meal agar (CMA, Shanghai yiyan bio-technology Co., Ltd., China). Cultures on PDA slants were transferred to PDA and CMA plates and incubated at 25°C for 7 days. Reverse colony pigmentation of strains grown on PDA and CMA was assessed according to Kornerup and Wanscher (1978). For morphological evaluation, microscope slides were prepared by placing mycelia from the cultures on PDA and CMA blocks (5 mm diameter) and then overlaid with a coverslip. The sizes and shapes of the microcharacteristics (e.g., ascomata, asci, ascospores, conidiogenous cells, and conidia) were determined using a light microscope (CX40, Olympus Corporation, Tokyo, Japan) and a scanning electron microscope (Quanta 200 FEG, FEI Company, Hillsboro, United States). Individual length and width measurements were taken for 30–100 replicates, including the absolute minima and maxima.

### DNA extraction, polymerase chain reaction, and sequencing

Specimens and live axenic cultures were prepared for DNA extraction. Genomic DNA was extracted using the Genomic DNA Purification Kit (Qiagen GmbH, Hilden, Germany) according to the manufacturer’s protocol. The primer pair ITS5 and ITS4 was used to amplify the nuclear ribosomal internal transcribed spacer region (ITS) (White et al., 1990). For amplification of the nuclear ribosomal large subunit (nr*LSU*) and the β-tubulin (*TUB2*) gene, PCR primer pairs LR5/LR0R and T1/Bt2b (Vilgalys and Hester, 1990; Rehner and Samuels, 1994; Glass and Donaldson, 1995; O’Donnell and Cigelnik, 1997) were employed. The translation elongation factor 1α (*TEF1*) gene was amplified using the primer pair EF1-688F/EF1-1251R (Alves et al., 2008). All of the PCR reactions were performed in a final volume of 50 μL containing 25 μL 2 × Taq PCR Master Mix (Tiangen Biotech Co., LTD, China), 0.5 μL of each primer (10 μM), 1 μL of genomic DNA, and 23 μL of RNase-Free water. PCR products were sequenced by Beijing Sinogenomax Co. Ltd., China.

### Phylogenetic analyses

Phylogenetic analyses were based on the nr*LSU* and combined ITS+nr*LSU* + *TUB2* + *TEF1* sequences. Sequences of ITS, nr*LSU*, *TUB2*, and *TEF1* were retrieved from GenBank and combined with those generated in our study. The taxonomic information and GenBank accession numbers are provided in Table 1. Sequences were aligned using Clustal X 2.0 and MEGA v6.06 software (Larkin et al., 2007; Tamura et al., 2013). After alignment, the sequences of the genes were concatenated. Conflicts among the six genes were tested using PAUP* 4.0b10 (Swofford, 2002). The results showed that the phylogenetic signals for the four loci were congruent (*p* = 0.03). Phylogenetic analyses were conducted using the Bayesian Inference (BI) and the Maximum Likelihood (ML) methods employing MrBayes v3.1.2 and RAxML 7.0.3 (Ronquist and Huelsenbeck, 2003; Stamatakis et al., 2008). Models of sequence evolution were estimated using jModelTest version 2.1.4 (Darriba et al., 2012). The following models were implemented in the Bayesian phylogenetic analyses: GTR + I + G for ITS and nr*LSU*, K80 + G for *TUB*, SYM + G for *TEF1*. The BI analysis was run on MrBayes v3.1.2 for 5 million generations. GTR + I was selected as the optimal model for ML analysis, and 1,000 rapid bootstrap replicates were performed on the dataset. Furthermore, ML analysis was applied to single-locus genealogies for ITS, nr*LSU*, *TUB2*, and *TEF1*.

**Table 1:** Specimen information and GenBank accession numbers for sequences used in this study.

Taxon	Voucher Info.^1^	Host/substrate	Locality	GenBank accession number				References
				ITS	nr*LSU*	*TUB2*	*TEF1*	
*Clonostachys agrawalii*	CBS 533.81	Decomposing buffalo horn	India	AF358241		AF358187		Schroers (2001)
*Clonostachys apocyni*	CBS 130.87^T^	Dead stem of *Apocynum cannabinum*	United States	AF210688		AF358168		Schroers (2001)
*Clonostachys aranearum*	GZAC QLS0625clo^T^	Spider	China	KU173835		KU212401		Chen et al. (2016)
*Clonostachys aureofulvella*	CBS 195.93	Root of tree	New Zealand	AF358226		AF358181		Schroers (2001)
*Clonostachys aureofulvella*	CBS 200.93	Bark of *Polylepis sericea*	Venezuela			AF358182		Schroers (2001)
*Clonostachys buxi*	CBS 696.93	Leaves of *Buxus sempervirens*	France	KM231840	KM231721	KM232111	KM231977	Lombard et al. (2015)
*Clonostachys byssicola*	CBS 364.78^T^	Wood	Venezuela	MH861151	MH872912	AF358153	KX184967	Schroers (2001), Moreira et al. (2016), andVu et al. (2019)
*Clonostachys byssicola*	CML 2309	*Fragaria ananassa*	Brazil	KC806269	KC806269	KF871149	KX184966	Abreu et al. (2014) and Moreira et al. (2016)
*Clonostachys candelabrum*	CBS 504.67	Soil	Netherlands	AF210668		KF871189	KX185029	Schroers (2001), Abreu et al. (2014), and Moreira et al. (2016)
*Clonostachys candelabrum*	CML 2313	Soil	Brazil	KC806296	KC806296	KF871186		Abreu et al. (2014)
*Clonostachys capitata*	CBS 218.93	Bark	Japan	MH862394	MH874054	AF358188		Schroers (2001) and Vu et al. (2019)
*Clonostachys chlorina*	CBS 287.90^T^	Brazil	Soil	MH862212	MH873895			Vu et al. (2019)
*Clonostachys chloroleuca*	CBS 141588^T^	Native soil from Cerrado	Brazil	KC806286	KC806286	KF871172	KX184988	Abreu et al. (2014) and Moreira et al. (2016)
*Clonostachys chloroleuca*	CBS 141589	Native soil from Cerrado	Brazil	KC806277	KC806277	KF871173	KX184978	Abreu et al. (2014) and Moreira et al. (2016)
*Clonostachys chuyangsinensis*	YFCC 895	Soil	China	MW199068	MW199057	MW201675	MW295968	This work
*Clonostachys chuyangsinensis*	YHH 896	Spider	Vietnam	MW199066	MW199055	MW201673	MW295966	This work
*Clonostachys chuyangsinensis*	YFCC 896^T^	Spider	Vietnam	MW199067	MW199056	MW201674	MW295967	This work
*Clonostachys coccicola*	BUcCo	*Unaspis citri*	Australia	KU720552	KU720550			Dao et al. (2016)
*Clonostachys coccicola*	BUcS	*Unaspis citri*	Australia	KU720551	KU720549			Dao et al. (2016)
*Clonostachys compactiuscula*	CBS 913.97	Bark of dead *Fagus* sp	United States	AF358245		AF358194		Schroers (2001)
*Clonostachys compactiuscula*	CBS 919.97	Twigs of *Acer* sp	United States	AF210690	AF210690			Schroers (2001)
*Clonostachys compactiuscula*	YFCC 894	Soil	China	MW291598	MW291602	MW295976	MW295971	This work
*Clonostachys compactiuscula*	YFCC 897	Soil	China	MW199071	MW199060	MW201678	MW295972	This work
*Clonostachys divergens*	CBS 967.73b^T^	Soil	Germany	AF210677	AF210677	AF358191		Schroers (2001)
*Clonostachys epichloë*	CBS 101037	*Sasa* sp	Japan	AF210675	AF210675	AF358209		Schroers (2001)
*Clonostachys eriocamporesiana*	MFLUCC 17-2620^T^	Dead stems of *Chromolaena odorata*	Thailand	MN699132		MN699965	MN699964	Hyde et al. (2020)
*Clonostachys eriocamporesii*	MFLUCC 19-0486^T^	Dead stems of *Pennisetum polystachion*	Thailand	MN699133	MN699128			Hyde et al. (2020)
*Clonostachys grammicospora*	CBS 209.93^T^	Standing dead tree	French Guiana	AF210678	MH874052	AF358206		Schroers (2001) and Vu et al. (2019)
*Clonostachys grammicosporopsis*	CBS 115.87	Bark of *Metrosideros* sp	New Zealand	AF210679	AF210679	AF358204		Schroers (2001)
*Clonostachys impariphialis*	HMAS 275560	Rotten bark	China	KX096609	KX096606			Zeng and Zhuang (2017)
*Clonostachys indicus*	IBP 2	Dead twigs of *Ficus virens*	India	KT291441				Prasher and Chauhan (2017)
*Clonostachys intermedia*	CBS 508.82^T^	Agricultural soil	Netherlands	AF210682		AF358205		Schroers (2001)
*Clonostachys intermedia*	KUC21274	Soil	South Korea	MH168099				Unpublished
*Clonostachys kowhai*	CBS 461.95^T^	Bark of *Sophora microphylla*	New Zealand	AF358250		AF358170		Schroers (2001)
*Clonostachys krabiensis*	MFLUCC 16-0254^T^	Dead leaves of *Pandanus* sp	Thailand	MH388335	MH376707			Tibpromma et al. (2018)
*Clonostachys kunmingensis*	YFCC 898^T^	Soil	China	MW199069	MW199058	MW201676	MW295969	This work
*Clonostachys kunmingensis*	YFCC 892	Soil	China	MW199070	MW199059	MW201677	MW295970	This work
*Clonostachys kunmingensis*	YFCC 967	Soil	China	OP023125	OP023116			This work
*Clonostachys levigata*	CBS 948.97	Branch of dead *Buxus sempervirens*	France	AF210680	AF210680	AF358196		Schroers (2001)
*Clonostachys lucifer*	CBS 100008	Bark of dead *Casearia arborea*	United States	AF210683		AF358208		Schroers (2001)
*Clonostachys moreaui*	CLL19024^T^	Bark of *Laurus novocanariensis*	Portugal		MT160524			Lechat et al. (2020)
*Clonostachys oblongispora*	CBS 100285^T^	Bark of dying tree of *Orixa japonica*	Japan	AF358248		AF358169		Schroers (2001)
*Clonostachys parva*	CBS 997.69^T^	Agricultural soil	Netherlands	AF210674	AF210674	AF358210		Schroers (2001)
*Clonostachys phyllophila*	CBS 921.97^T^	Leaves, Fallen plant	France	AF210664	AF210664			Schroers (2001)
*Clonostachys pilosella*	BRFM 3113^T^	Bark	French Guiana		MT248415			Lechat and Fournier (2020)
*Clonostachys pityrodes*	CBS 102033	Bark	Mauritius	AF210672	AF210672	AF358212		Schroers (2001)
*Clonostachys pityrodes*	CBS 126394	Small, standing dead tree	Sri Lanka	MH864280	MH875729			Vu et al. (2019)
*Clonostachys pnagiana*	BRFM 3057^T^	Bark	French Guiana		MT248416			Lechat and Fournier (2020)
*Clonostachys pseudochroleuca*	CBS 187.94^T^	Base of decaying palm frond	French Guiana	KJ499909	KJ499909	KF871188	KX185003	Abreu et al. (2014) and Moreira et al. (2016)
*Clonostachys pseudochroleuca*	CML 1982	Soil	Brazil	KC806263	KC806263	KF871165	KX185002	Abreu et al. (2014) and Moreira et al. (2016)
*Clonostachys pseudostriata*	CBS 119.87	Bark	Indonesia	AF358251		AF358183		Schroers (2001)
*Clonostachys pseudostriatopsis*	MAFF 239827	Bark of fallen twigs	Japan			AB237465		Hirooka and Kobayashi (2007)
*Clonostachys ralfsii*	CBS 129.87	Bark	New Zealand	AF210676	AF210676	AF358195		Schroers (2001)
*Clonostachys rhizophaga*	CBS 202.37	Root of *Ulmus americana*	United States	AF358225	MH867396	AF358156		Schroers (2001) and Vu et al. (2019)
*Clonostachys rhizophaga*	CBS 361.77	Culture contaminant	Switzerland	AF358228		AF358158	KX184993	Schroers (2001)
*Clonostachys rhizophaga*	CML 2312	Culture contaminant	Brazil	KC806275	KC806275	KF871157	KX184992	Abreu et al. (2014) and Moreira et al. (2016)
*Clonostachys rhizophaga*	YFCC 900	Soil	China	MW199074	MW199063	MW201681	MW295974	This work
*Clonostachys rogersoniana*	CBS 582.89	Rain forest soil	Brazil	AF210691		AF358189		Schroers (2001)
*Clonostachys rogersoniana*	CML 1216	Soil	Brazil	KC806287	KC806287	KF871178	KX185017	Abreu et al. (2014) and Moreira et al. (2016)
*Clonostachys rogersoniana*	YFCC 899	Soil	China	MW199073	MW199062	MW201680	MW295973	This work
*Clonostachys rosea*	CBS 154.27	Soil	United States	MH854911	MH866405	AF358160	KX184995	Schroers (2001), Moreira et al. (2016), and Vu et al. (2019)
*Clonostachys rosea*	CBS 406.95	Bark of *Salix* sp	France	AF358249		AF358167		Schroers (2001)
*Clonostachys rosea*	CBS 710.86^T^	Soil	Netherlands	AF358235	MH873700	AF358161	KX184999	Schroers (2001), Moreira et al. (2016), and Vu et al. (2019)
*Clonostachys rosea*	CML 2310	*Fragaria ananassa*	Brazil	KC806257	KC806257	KF871146	KX184998	Abreu et al. (2014) and Moreira et al. (2016)
*Clonostachys rosea*	YFCC 893	Soil	Thailand	ON287194	ON303656	ON314171	ON314172	This work
*Clonostachys rossmaniae*	CBS 210.93	Bark of twigs	French Guiana	AF358227		AF358213		Schroers (2001)
*Clonostachys samuelsii*	CBS 699.97	Bark	Venezuela	AF358236		AF358190		Schroers (2001)
*Clonostachys samuelsii*	CBS 700.97	Bark	United States	AF210689				Schroers (2001)
*Clonostachys saulensis*	BRFM 2782^T^	Bark of dead liana	French Guiana	MK635054				Lechat et al. (2019)
*Clonostachys setosa*	CBS 834.91	Twig	Cuba	AF210670	AF210670	AF358211		Schroers (2001)
*Clonostachys sesquicillii*	CBS 180.88	Twigs and lichen	Guyana	AF210666	AF210666	AF358214		Schroers (2001)
*Clonostachys solani*	CBS 183.30	Garden soil	Netherlands	MH855105	MH866555	AF358222		Schroers (2001), Vu et al. (2019)
*Clonostachys solani*	CBS 223.72b	Wheat field soil	Germany	MH860460	MH872186	AF358223		Schroers (2001), Vu et al. (2019)
*Clonostachys solani*	CBS 697.88	Bark	Germany	MH862150	MH873842	AF358216		Schroers (2001), Vu et al. (2019)
*Clonostachys solani*	CBS 752.68	Wood of angiosperm tree	Germany	MH859224	MH870947	AF358221		Schroers (2001), Vu et al. (2019)
*Clonostachys solani*	YFCC 901	Soil	China	MW199072	MW199061	MW201679	MW295975	This work
*Clonostachys spinulosispora*	CBS 133762^T^	Leaves	French Guiana	MH634702	KY006568			Lechat and Fournier (2018)
*Clonostachys sporodochialis*	CBS 101921^T^	Bark	United States	AF210685		AF358149		Schroers (2001)
*Clonostachys sporodochialis*	CLL-GUY-12-046	Bark	French Guiana	KJ802125				Crous et al. (2014)
*Clonostachys subquaternata*	CBS 107.87	Wood	Venezuela			AF358207		Schroers (2001)
*Clonostachys vesiculosa*	HMAS 183151^T^	Decaying leaves of a dicotyledonous plant	China	HM050304	HM050302			Luo and Zhuang (2010)
*Clonostachys viticola*	MUM 18.51^T^	root of *Vitis vinifera*	Peru	MK156282		MK156290	MK156286	Torcato et al. (2020)
*Clonostachys viticola*	CAA 945	root of *Vitis vinifera*	Peru	MK156283		MK156291	MK156287	Torcato et al. (2020)
*Clonostachys wenpingii*	HMAS 172156^T^	Dead leaves	China	EF612465	HM042410	HM054127	HM054097	Zhao et al. (2011)
*Clonostachys zelandiaenovae*	CBS 232.80	Wood	New Zealand	AF210684	AF210684	AF358185		Schroers (2001)
*Stanjemonium grisellum*	CBS 655.79^T^	Soil	United States	AY632671	MH873004	AY632687		Zuccaro et al. (2004) and Vu et al. (2019)
*Stanjemonium ochroroseum*	CBS 656.79^T^	Soil	United States	AY632672	AF049172	AY632688	AF049194	Zuccaro et al. (2004)

1 CAA, Culture collection of Artur Alves, housed at Department of Biology, University of Aveiro, Portugal; CBS, Westerdijk Fungal Biodiversity Institute, Utrecht, The Netherlands; CML, Coleção Micológica de Lavras, Universidade Federal de Lavras, Lavras, Minas Gerais, Brazil; GZAC, Institute of Fungus Resources, Guizhou University, Guiyang, China; HMAS, Herbarium of Mycology, Institute of Microbiology, Chinese Academy of Sciences, Beijing, China; MFLUCC: Mae Fah Luang University Culture Collection, Chiang Rai, Thailand; MUM, Culture collection hosted at Center for Biological Engineering of University of Minho, Braga, Portugal; YFCC, Yunnan Fungal Culture Collection, Yunnan University, Kunming, China; YHH, Yunnan Herbal Herbarium, Yunnan University, Kunming, China. ^T^ex-type strain. Boldface: data generated in this study.

We applied a (phylo-) genetic distance matrix calculation for the markers (ITS, *TUB2*, and *TEF1*) to assess species boundaries of 14 *Clonostachys* spp. (Supplementary Tables S1–S3), because their sequence data for the three loci were complete. The pairwise genetic distances of the 14 *Clonostachys* lineages were measured based on the Kimura two-parameter model using MEGA v6.06 software (Tamura et al., 2013).

## Results

### Sequencing and phylogenetic analyses

**Figure 1:**
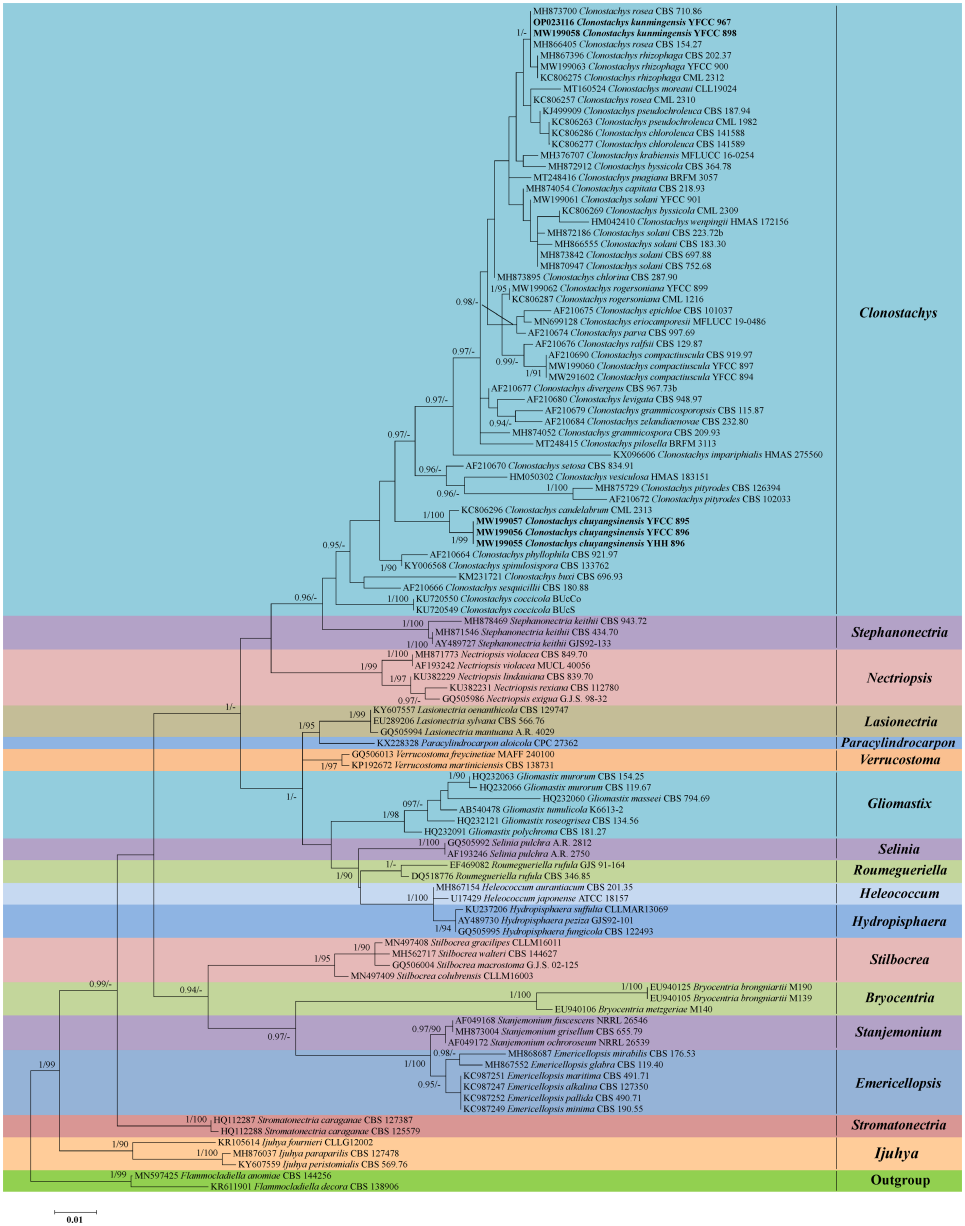
Phylogenetic reconstruction of *Clonostachys* and related genera in Bionectriaceae obtained from the nr*LSU* sequences based on Bayesian inference and Maximum Likelihood analyses. Statistical support values (≥0.9/90%) are shown at the nodes for BI posterior probabilities/ML bootstrap support. Materials in bold type are those analyzed in this study.

**Figure 2:**
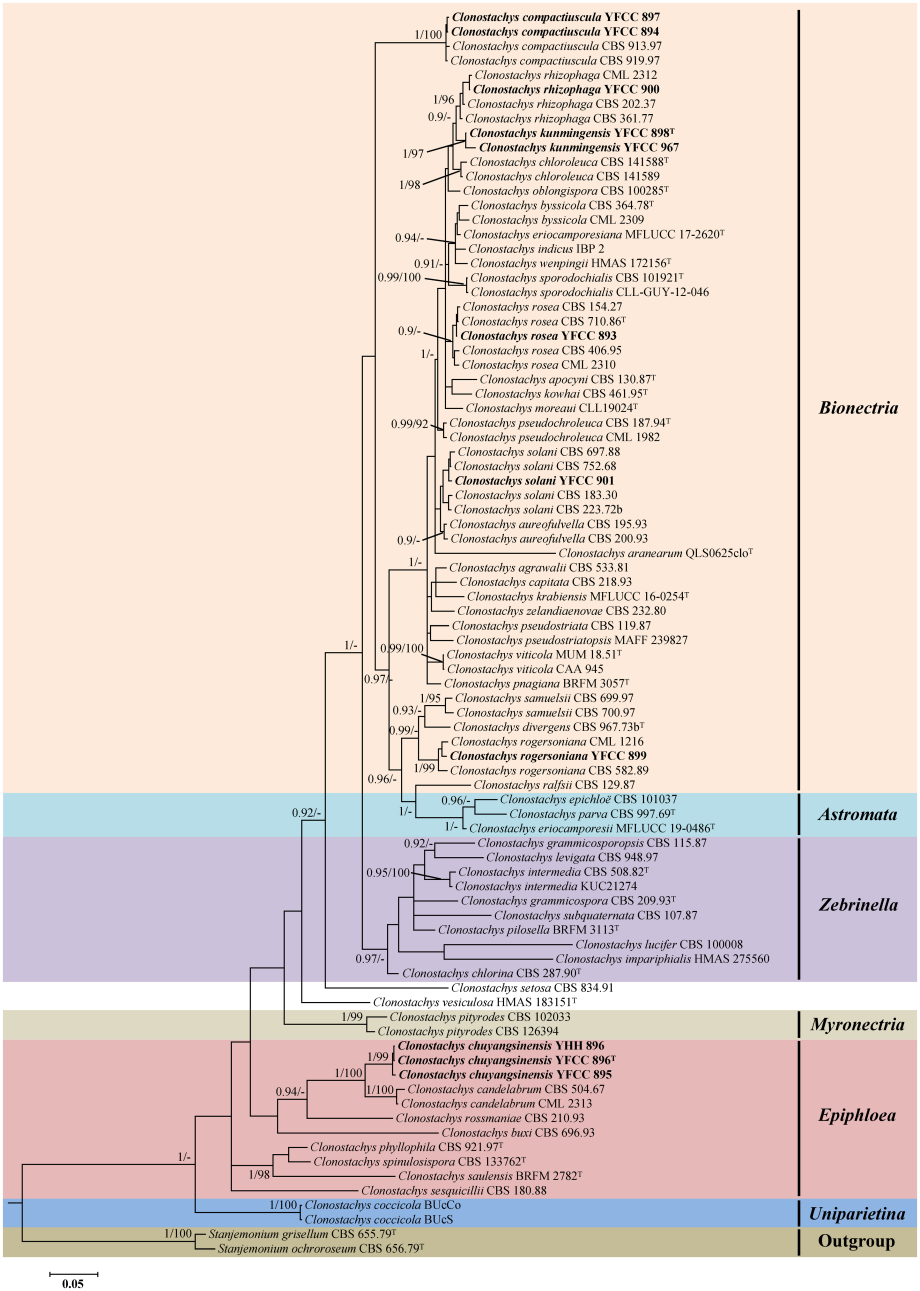
Phylogenetic tree of *Clonostachys* based on Bayesian inference and Maximum Likelihood analyses of a 4-locus (ITS, nr*LSU*, *TUB2*, and *TEF1*) dataset. Statistical support values (≥ 0.9/90%) are shown at the nodes for BI posterior probabilities/ML bootstrap support. Materials in bold type are those analyzed in this study. Isolates representing ex-type material are marked with “T.”

Phylogenetic analyses based on nr*LSU* data consisting of 107 fungal taxa confirmed the presence and positions of *Clonostachys* and related genera within Bionectriaceae. Eighteen well-supported clades were recognized based on both BI and ML analyses of the 107 taxa from Bionectriaceae and *Flammocladiella* (Flammocladiaceae, Hypocreales) that accommodate species of the genera *Bryocentria*, *Clonostachys*, *Emericellopsis*, *Gliomastix*, *Heleococcum*, *Hydropisphaera*, *Ijuhya*, *Lasionectria*, *Nectriopsis*, *Paracylindrocarpon*, *Roumegueriella*, *Selinia*, *Stanjemonium*, *Stephanonectria*, *Stilbocrea*, *Stromatonectria*, *Verrucostoma*, and *Flammocladiella* (Figure 1). The genus *Clonostachys* was phylogenetically clustered with *Stephanonectria*, and *Emericellopsis* had a close genetic relationship with *Stanjemonium*, but they were clearly distinguished from their allied genera by forming four separate clades in the family Bionectriaceae (Figure 1). The combined dataset included sequences from 86 fungal taxa (Table 1). The final dataset consisted of 2,900 bp of sequence data, including gaps (ITS, 654 bp; nr*LSU*, 903 bp; *TUB2*, 711 bp; and *TEF1*, 632 bp). Both BI and ML analyses produced trees with similar topologies that resolved most *Clonostachys* lineages in separate terminal branches (Figure 2). Phylogenetic trees inferred from analyses of combined data divided *Clonostachys* into six distinguished clades, designated as *Astromata*, *Bionectria*, *Epiphloea*, *Myronectria*, *Uniparietina*, and *Zebrinella* clades (Figure 2). The phylogenetic analyses suggested the existence of distinct species in the *Bionectria* and *Epiphloea* clade that we accordingly propose as new species: *C. chuyangsinensis*, which was found in the *Epiphloea* clade, and *C. kunmingensis*, which was found in the *Bionectria* clade (Figure 2).

The tree topologies for the individual loci (ITS, nr*LSU*, *TUB2*, and *TEF1*) did not show congruence (Supplementary Figures S1–S4). However, in all analyses *C. chuyangsinensis* had a close genetic relationship with *C. candelabrum*. *Clonostachys chloroleuca* and *C. rhizophaga* were sisters to the newly discovered species *C. kunmingensis*, although this relationship received significant bootstrap support only from ITS and *TUB2*. Phylogenetic analyses based on nr*LSU* data revealed that *C. kunmingensis* was closely related to *C. rosea* (Figure 1; Supplementary Figure S2). And the nr*LSU* sequences cannot distinguish the two species. But they were regarded as different species with strong support from ITS, *TUB2* and *TEF1* (Supplementary Figures S1, S3, S4).

The genetic divergence comparisons showed that: (1) the minimum thresholds (p-distances) to distinguish genetic species in the *Clonostachys* lineages were 0.005, 0.017, and 0.026 for ITS, *TUB2*, and *TEF1*, respectively (Supplementary Tables S1–S3); (2) the *TEF1* sequence data provided the best resolution distinguishing *Clonostachys* spp., followed by *TUB2* and ITS sequences (Supplementary Tables S1–S3); and (3) the genetic distances strongly supported recognition of *C. chuyangsinensis* and *C. kunmingensis* as two new taxa (Table 2).

**Table 2:** Genetic distance (*p*-distances) of the two new *Clonostachys* species with their related species.

Subgenus	Taxa	Marker		
		ITS	*TUB2*	*TEF1*
*Epiphloea*	*Clonostachys chuyangsinensis* – *Clonostachys candelabrum*	0.029	0.097	0.083
*Bionectria*	*Clonostachys kunmingensis* – *Clonostachys chloroleuca*	0.009	0.042	0.036
	*Clonostachys kunmingensis* – *Clonostachys rhizophaga*	0.012	0.023	0.058

## Taxonomy

In this study, a collection of 23 isolates of unknown identity were shown to represent five known species and two new species of *Clonostachys*. The phylogenetic positions of the five known species were evaluated according to phylogenetic inferences based on four loci (ITS, nr*LSU*, *TUB2*, and *TEF1*), including *C. compactiuscula*, *C. rhizophaga*, *C. rogersoniana*, and *C. solani* from China, and *C. rosea* from Thailand (see Table 1; Figure 2). The two new species, provided with the names *C. chuyangsinensis* from Vietnam and China and *C. kunmingensis* from China, were recognized based on morphological characteristics and molecular data.

**Figure 3:**
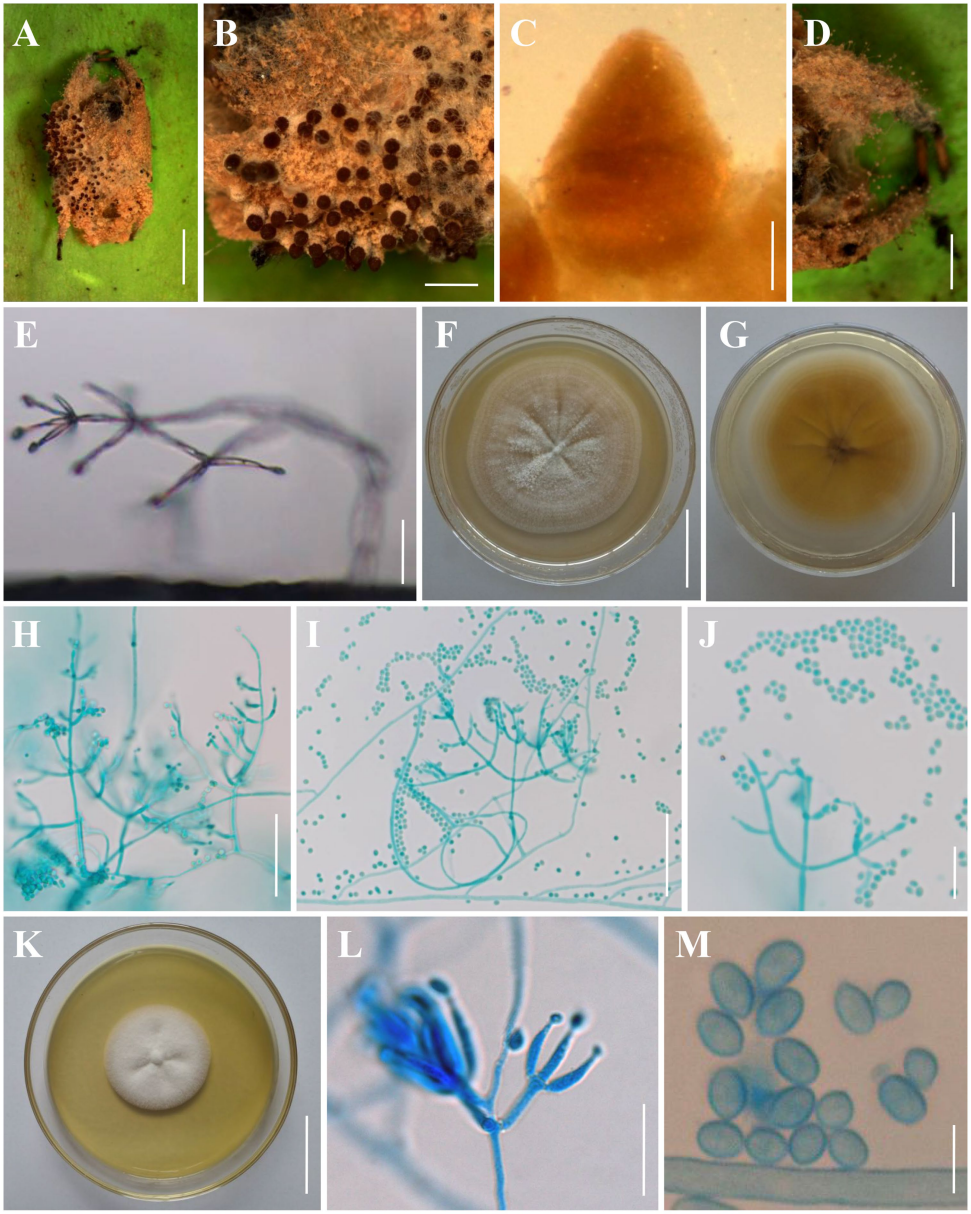
Morphology of *Clonostachys chuyangsinensis*. **(A)** Infected spider. **(B)** Ascomata on the host. **(C)** Front view of perithecium. **(D, E)** Conidiogenous structures on the host. **(F, G)** Colony obverse and reverse on PDA. **(H–J)** Conidiophores, conidiogenous cells, and conidia on PDA. **(K)** Colony obverse on CMA. **(L)** Conidiophores, conidiogenous cells, and conidia on CMA. **(M)** Conidia on CMA. Scale bars: **(A)** = 3 mm; **(B,D)** = 1 mm; **(C)** = 100 μm; **(E,J,L)** = 20 μm; **(F,G,K)** = 30 mm; **(H,I)** = 50 μm; **(M)** = 5 μm.

*Clonostachys chuyangsinensis* H. Yu & Y. Wang, sp. nov. Figure 3.

MycoBank number 843885.

Etymology: named after Chu Yang Sin National Park, where this species was first discovered.

Type: Vietnam, Dak Lak Province, Chu Yang Sin National Park (12°29’N, 108°43′E, 1659 m above sea level), on a spider on the underside of a leaf, October 22, 2017, collected by Yuan-Bing Wang (holotype: YHH 896; ex-type: YFCC 896).

Description: **Sexual morph:** Ascomata on a brown spider, perithecial, solitary or densely crowded in groups, subglobose to oval, (280–)290–380(−400) × (240–)260–330(−340) μm (n = 30), collapsing laterally when dry, pale brown when fresh, becoming dark brown to nearly black when dry, not changing color in 3% KOH or in lactic acid; surface smooth. Asci and ascospores not observed. **Asexual morph:** Infected spider host covered with a dense brown mycelial mat. Hyphae branched, septate, hyaline, smooth. Conidiophores verticillium-like; phialides divergent in whorls of 2–5 or single from lower levels, generally slightly tapering toward the tip, (5–)5.6–28.3(−36) × (1–)1.4–3.6(−4) μm (n = 30). Conidia smooth-walled, hyaline, subglobose to ellipsoid, (2–)2.5–4.6(−4.8) × (2–)2.4–3.5(−4) μm (n = 30). Colonies on PDA reached 28–32 mm in diameter after 7 days at 25°C, white, circular; reverse pale to light orange (5–6A3–4). Colony surface white powdery to granulose because of the conidiophores and conidial masses; aerial mycelium sparsely produced or absent. Conidiophores monomorphic, verticillate, arising from the agar surface or from the sparse aerial mycelium; stipes (20–)40–130(−150) μm long, (2–)2.5–4(−5) μm wide at the base (n = 50); primary branches divergent, forming independent side-branches; terminal branches and phialides divergent or adpressed; terminal phialides flask-shaped, or cylindrical but narrowing in the upper part, (4.5–)5.5–44.2(−60) × (1.2–)1.5–3.8(−4) μm (n = 50). Conidia in white imbricate columns, smooth-walled, hyaline, subglobose to ellipsoid, (2.2–)2.4–4.7(−5) × (1.5–)1.8–3.5(−3.8) μm (n = 50). Setae not observed. Colonies on CMA reached 25–30 mm in diameter after 7 days at 25°C, white, circular; reverse pale yellowish (1-2A3). Colony surface white powdery due to conidial masses, cottony to felty due to aerial mycelium. Conidiophores monomorphic, verticillate, arising from the agar surface or from the sparse aerial mycelium; stipes (20–)30–145(−160) μm long, (2–)2.5–4(−4.5) μm wide at the base (n = 50); primary branches divergent, forming independent side-branches; terminal branches and phialides divergent or adpressed; terminal phialides flask-shaped, or cylindrical but narrowing in the upper part, (4.5–)5.5–44.2(−50) × (1.2–)1.5–3.8(−4.2) μm (n = 50). Conidia in white imbricate columns, smooth-walled, hyaline, subglobose to ellipsoid, ovoid, (2–)2.2–5(−5.5) × (1.5–)2–3.5(−4) μm (n = 50). Setae not observed.

*Distribution*: Chu Yang Sin National Park, Dak Lak Province, Vietnam; Kunming City, Yunnan Province, China.

Additional materials examined: China, Yunnan Province, Kunming City, Wild Duck Forest Park (25°13’N, 102°87′E, 2100 m above sea level), from soil on the forest floor, August 20, 2018, Yao Wang (living culture: YFCC 895); China, Yunnan Province, Kunming City, Songming County, Dashao Village (25°24’N, 102°55′E, 2697 m above sea level), from *Ophiocordyceps highlandensis*, August 25, 2018, De-Xiang Tang (living culture: YFCC 8591) (Zhao et al., 2021).

Notes: Morphologically, *C. chuyangsinensis* resembles the phylogenetically sister species *C. candelabrum*. The shape and size of the conidia and the colony color of *C. chuyangsinensis* among other morphological features have been observed in *C. candelabrum*. However, *C. chuyangsinensis* can be distinguished from *C. candelabrum* by its long phialides ((4.5–)5.5–44.2(−50) × (1.2–)1.5–3.8(−4.2) μm). Both morphological study and phylogenetic analyses of combined ITS, nr*LSU*, *TUB2*, and *TEF1* sequence data support that this fungus is a distinct species in the genus *Clonostachys*.

**Figure 4:**
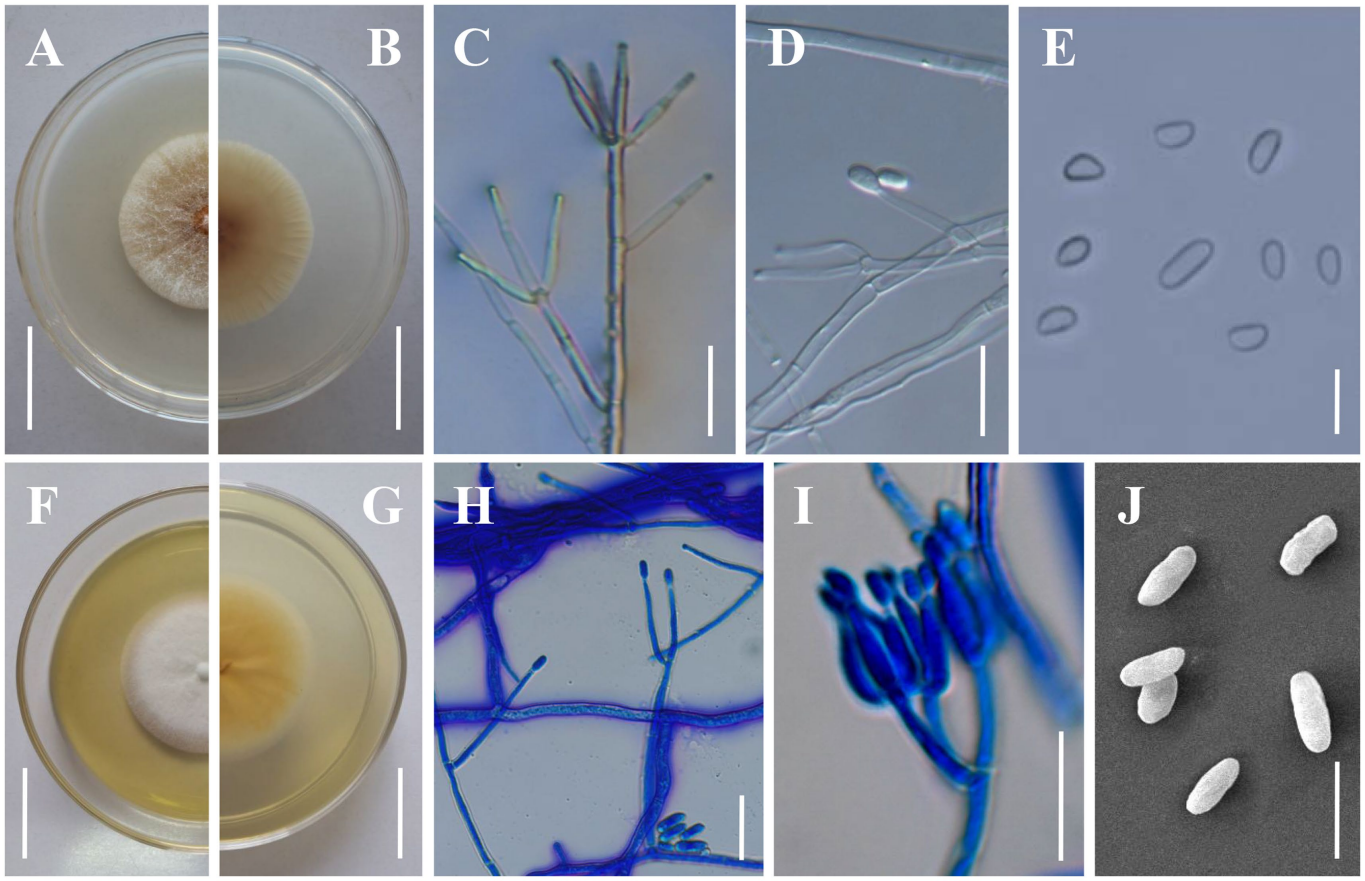
Morphology of *Clonostachys kunmingensis*. **(A,B)** Colony obverse and reverse on PDA. **(C,D)** Verticillium-like primary conidiophores on PDA. **(E)** Conidia from secondary conidiophores. **(F,G)** Colony obverse and reverse on CMA. **(H)** Verticillium-like primary conidiophores on CMA. **(I)** Secondary conidiophores on CMA. **(J)** Conidia from primary conidiophores. Scale bars: **(A,B,F,G)** = 20 mm; **(C,D,H,I)** = 20 μm; **(E,J)** = 10 μm.

*Clonostachys kunmingensis* H. Yu & Y. Wang, sp. nov. Figure 4.

MycoBank number 843886.

Etymology: named after the location Kunming City where the species was collected.

Type: China, Yunnan Province, Kunming City, Wild Duck Forest Park (25°13’N, 102°87′E, 2100 m above sea level), from soil on the forest floor, August 10, 2019, Yao Wang (holotype: YHH 898, dried specimen; ex-type: YFCC 898).

Description: **Sexual morph:** Undetermined. **Asexual morph:** Colonies on PDA reaching 32–35 mm in diameter after 7 days at 25°C, pale yellow (4A2–3), circular; reverse pale orange (5A2–3). Colony surface cottony to felty due to aerial mycelium. Conidiophores dimorphic. Primary conidiophores verticillium-like, arising from the agar surface or from the sparse aerial mycelium; (80–)120–260(−380) μm high, stipes (20–)60–140(−230) μm long, (2–)3.5–5(−5.5) μm wide at the base (n = 50), sometimes with short side branches arising from the upper part; phialides divergent, in whorls of 2–6, sometimes singly from lower levels, (14.2–)19.1–36.4(−52.6) × (2–)2.5–3.5(−3.9) μm (n = 50), straight, cylindrical, slightly tapering toward the tip. Secondary conidiophores penicillate, solitary to gregarious, with divergent branching penicilli; bi-to quarter-verticillate, (15–)30–100(−125) μm long, (3–)3.5–5(−5.5) μm wide at the base (n = 50); penicillus 90–145 μm high, typically with two primary branches, divergent, terminating in moderately divergent metulae and adpressed phialides; phialides divergent or adpressed, in whorls of 2–6, almost cylindrical tapering in the upper part, straight to slightly curved, (5.6–)8.0–17.5(−25) μm long, (2–)2.5–3.2(−4) μm wide at the base, (1–)1.2–1.4(−1.6) μm wide near the aperture (n = 50); intercalary phialides rarely observed. Conidial masses on verticillium-like conidiophores small and round collapsing to form whitish, watery masses; conidial masses on penicillate conidiophores inconspicuous, short, and rather thick, columnar, white. Conidia from secondary conidiophores slightly curved, with one slightly flattened side, distally broadly rounded, with laterally displaced hila, (4–)4.2–8.5(−9) × (2.2–)2.5–4(−4.5) μm (n = 100), held in imbricate; conidia from primary conidiophores larger, oblong to cylindrical, frequently less curved, sometimes without a visible hilum, (6–)6.7–11.2(−14) × (2–)2.3–4.5(−4.8) μm (n = 100). Colonies on CMA reaching 25–32 mm in diameter after 7 days at 25°C, white, circular; reverse yellowish white to light yellow (4A2–5). Colony surface white powdery due to conidial masses. Aerial mycelium on CMA not thick, on PDA strongly developed in thick, often erect hyphal strands. Size and shape of Conidiophores, phialides and conidia similar on PDA and CMA.

Additional materials examined: China, Yunnan Province, Kunming City, Songming County, Dashao Village (25°24’N, 102°55′E, 2750 m above sea level), from soil on the forest floor, August 24, 2019, Yao Wang (living culture: YFCC 892, 967).

Notes: Regarding phylogenetic relationships, *C. kunmingensis* is closely related to *C. rhizophaga* and *C. chloroleuca* and further grouped with *C. oblongispora* (Figure 2). However, *C. kunmingensis* can be distinguished from *C. rhizophaga* and *C. chloroleuca* by its oblong to cylindrical conidia ((6–)6.7–11.2(−14) × (2–)2.3–4.5(−4.8) μm). *Clonostachys kunmingensis* consistently showed unpigmented conidial masses, while conidial masses of *C. rhizophaga* and *C. chloroleuca* can be greenish or weakly greenish (Moreira et al., 2016). *Clonostachys oblongispora* differs from *C. kunmingensis* by its longer conidia ((9–)12.6–13.6–14(−19.8) × (2.6–)3.2–3.6–3.8(−4.2) μm) (Schroers, 2001). Morphologically, *C. kunmingensis* is similar to *C. rosea* in terms of the shape and size of the conidiogenous cells and the shape of the conidia (Schroers, 2001). However, our morphological observation revealed some differences between them. Colonies of *C. kunmingensis* on PDA are pale yellow whereas those of *C. rosea* are white. Furthermore, conidia from secondary conidiophores of *C. kunmingensis* ((4–)4.2–8.5(−9) × (2.2–)2.5–4(−4.5) μm) are larger than those of *C. rosea* ((4.2–)4.8–5.2–5.6(−6.6) × (2–)2.4–2.8–3(−3.4) μm).

## Discussion

*Clonostachys* species are widely distributed and occupy diverse habitats, with various host/substrate associations (see Table 1). The species distribution is cosmopolitan, with the height of known species diversity occurring in tropical regions; the habitat diversity is complicated, with most of the known species having unspecific saprotrophic ability (Schroers, 2001). These known species are commonly found in soils, litter, and dead plant substrata as saprotrophs. They have also been reported as endophytes and epiphytes of living plants (Torcato et al., 2020). Another aspect of the biology of *Clonostachys* species is their unspecific parasitic ability. Some *Clonostachys* spp. are known as destructive mycoparasites, with *C. rosea* and *C. rosea* f. *catenulata* being used as biocontrol agents against various ascomycetes, soil-borne hyphomycetes, and basidiomycetes (Schroers, 2001; Chatterton et al., 2008). They are also parasitic to myxomycetes, nematodes, ticks, mollusks, and leafhoppers (Schroers, 2001; Toledo et al., 2006). In this study, we described a novel species, *C. chuyangsinensis*, which was isolated from a large spider. In fact, *Clonostachys* species parasitic on spiders have rarely been reported, apart from *C. aranearum* (Chen et al., 2016). The present study provides new evidence for *Clonostachys* sp. as an araneopathogenic fungus, thus extending our knowledge of the occurrence and distribution of spider-pathogenic fungi.

Compared with the anamorph of *Clonostachys* with simple morphological architectures, the teleomorph provided more valuable morphological information to recognize individual *Clonostachys* species. Schroers (2001) classified the teleomorph in the six distinguished subgenera *Astromata*, *Bionectria*, *Epiphloea*, *Myronectria*, *Uniparietina*, and *Zebrinella* based on stroma morphology, stroma-perithecium wall interface structure, perithecial wall anatomy, habit of the perithecia on the natural substratum, and ascospore ornamentation and septation. Our phylogenetic analyses based on the combined ITS+nr*LSU* + *TUB2* + *TEF1* sequences provide additional evidence supporting these morphologically delimited subgenera (Figure 2). It seems that the divisions of six subgenera do not contradict the unity of the entire genus *Clonostachys*. All taxa of six subgenera are united by the phenotypic characteristics of the anamorph such as penicillate conidiophores, conidia held in imbricate columns, and predominantly more or less curved conidia with mostly laterally displaced hila (Schroers, 2001). Some intraspecific variations in conidiomata, intercalary phialides, conidiophore dimorphism, and conidial mass color have hampered species identification in *Clonostachys*, but to a certain extent these may reflect subgeneric affinities (Schroers, 2001). In the current study, it should be noted that the phylogenetic trees inferred from the analyses of combined data excluded *C. setosa* and *C. vesiculosa* from the six subgenera (Figure 2). The two species should belong to the subgenus *Epiphloea* based on diagnostic features (Schroers, 2001; Luo and Zhuang, 2010). However, they are distant relatives of *Epiphloea* spp. from our results (Figure 2). The phenotypic similarities among non-sister species may result from convergent morphological evolution, perhaps due to occupation of similar ecological niches (Bischoff et al., 2009). Therefore, we propose to protect *Clonostachys* as the genus name for the entire clade, while acknowledging that future studies including more data and taxonomic sampling may introduce new genera to accommodate these subgenera.

The multilocus phylogenetic approach taken in this study of the genus *Clonostachys* has shed considerable light on this important group of fungi. The results of the present work indicate that the nr*LSU* sequences provided little valuable information to separate *Clonostachys* spp., although they were conducive to determining the phylogenomic relationships between *Clonostachys* and its related genera. In contrast, sequence data for the ITS and protein-coding gene region *TUB2* provided good resolution of *Clonostachys* spp., confirming the results of previous studies (Schroers, 2001; Hirooka and Kobayashi, 2007; Luo and Zhuang, 2010; Chen et al., 2016; Prasher and Chauhan, 2017). Our study also introduced sequence data for the *TEF1* gene region. This region requires only two primers and is easily amplified. Although the sequence length of the *TEF1* fragment was the shortest among the four loci analyzed in this study, the introns within *TEF1* provided the greatest concentration of informative nucleotide variation and degree of phylogenetic resolution for terminal clades in *Clonostachys*. Additionally, the genetic distances of *Clonostachys* species for *TEF1* were significantly higher than those for ITS and *TUB2* (Supplementary Tables S1–S3). Future studies will determine the use of this single locus for the recognition and identification of phylogenetic species in *Clonostachys* and other fungal species.

## Data availability statement

The datasets presented in this study can be found in online repositories. The names of the repository/repositories and accession number(s) can be found in the article/Supplementary material.

## Author contributions

YW: conceptualization. YW: methodology, writing—original draft preparation, and formal analysis. YW and RL: software. D-XT and RL: validation. YW, D-XT, Y-BW, CT, and HY: investigation. YW, D-XT, and V-MD: resources. HY: writing—review and editing and funding acquisition. All authors reviewed and approved the final manuscript.

## Funding

This work was supported by the National Natural Science Foundation of China (Nos 32200013 and 32160005).

## Conflict of interest

The authors declare that the research was conducted in the absence of any commercial or financial relationships that could be construed as a potential conflict of interest.

## Publisher’s note

All claims expressed in this article are solely those of the authors and do not necessarily represent those of their affiliated organizations, or those of the publisher, the editors and the reviewers. Any product that may be evaluated in this article, or claim that may be made by its manufacturer, is not guaranteed or endorsed by the publisher.

## Footnotes

^1^ www.ncbi.nlm.nih.gov

^2^ https://www.ncbi.nlm.nih.gov/taxonomy
