# Supplementary material for: Phylogeny and systematics of the genus Clonostachys
Source: Front Microbiol. 2023 Mar 3;14:1117753. doi: 10.3389/fmicb.2023.1117753 (PMC10020229; doi:10.3389/fmicb.2023.1117753)
Supplement: Supplementary file 1 [file Data_Sheet_1.docx]

**TABLE S1** Pairwise genetic distance matrix of *Clonostachys* species for ITS sequences

| Taxon | 1 | 2 | 3 | 4 | 5 | 6 | 7 | 8 | 9 | 10 | 11 | 12 | 13 | 14 |
| --- | --- | --- | --- | --- | --- | --- | --- | --- | --- | --- | --- | --- | --- | --- |
| 1 (*C. buxi* CBS 696.93) |  |  |  |  |  |  |  |  |  |  |  |  |  |  |
| 2 (*C. byssicola* CBS 364.78) | 0.131 |  |  |  |  |  |  |  |  |  |  |  |  |  |
| 3 (*C. candelabrum* CBS 504.67) | 0.110 | 0.089 |  |  |  |  |  |  |  |  |  |  |  |  |
| 4 (*C. chloroleuca* CBS 141588) | 0.152 | 0.019 | 0.097 |  |  |  |  |  |  |  |  |  |  |  |
| 5 (*C. chuyangsinensis* YHH 896) | 0.086 | 0.095 | 0.029 | 0.104 |  |  |  |  |  |  |  |  |  |  |
| 6 (*C. compactiuscula* YFCC 897) | 0.119 | 0.039 | 0.094 | 0.044 | 0.094 |  |  |  |  |  |  |  |  |  |
| 7 (*C. kunmingensis* YFCC 898) | 0.138 | 0.014 | 0.091 | 0.009 | 0.098 | 0.034 |  |  |  |  |  |  |  |  |
| 8 (*C. pseudochroleuca* CBS 187.94) | 0.138 | 0.012 | 0.088 | 0.012 | 0.094 | 0.036 | 0.007 |  |  |  |  |  |  |  |
| 9 (*C. rhizophaga* YFCC 900) | 0.141 | 0.017 | 0.091 | 0.012 | 0.098 | 0.042 | 0.012 | 0.005 |  |  |  |  |  |  |
| 10 (*C. rogersoniana* YFCC 899) | 0.119 | 0.039 | 0.088 | 0.045 | 0.089 | 0.019 | 0.039 | 0.037 | 0.042 |  |  |  |  |  |
| 11 (*C. rosea* YFCC 893) | 0.145 | 0.017 | 0.094 | 0.012 | 0.101 | 0.042 | 0.012 | 0.005 | 0.005 | 0.042 |  |  |  |  |
| 12 (*C. solani* YFCC 901) | 0.141 | 0.022 | 0.100 | 0.017 | 0.107 | 0.037 | 0.017 | 0.009 | 0.009 | 0.042 | 0.009 |  |  |  |
| 13 (*C. viticola* MUM 18.51) | 0.138 | 0.019 | 0.082 | 0.024 | 0.094 | 0.034 | 0.019 | 0.012 | 0.017 | 0.029 | 0.017 | 0.022 |  |  |
| 14 (*C. wenpingii* HMAS 172156) | 0.148 | 0.019 | 0.097 | 0.029 | 0.104 | 0.050 | 0.024 | 0.017 | 0.022 | 0.050 | 0.022 | 0.027 | 0.024 |  |

**TABLE S2** Pairwise genetic distance matrix of *Clonostachys* species for partial *TUB2* sequences

| Taxon | 1 | 2 | 3 | 4 | 5 | 6 | 7 | 8 | 9 | 10 | 11 | 12 | 13 | 14 |
| --- | --- | --- | --- | --- | --- | --- | --- | --- | --- | --- | --- | --- | --- | --- |
| 1 (*C. buxi* CBS 696.93) |  |  |  |  |  |  |  |  |  |  |  |  |  |  |
| 2 (*C. byssicola* CBS 364.78) | 0.171 |  |  |  |  |  |  |  |  |  |  |  |  |  |
| 3 (*C. candelabrum* CBS 504.67) | 0.173 | 0.203 |  |  |  |  |  |  |  |  |  |  |  |  |
| 4 (*C. chloroleuca* CBS 141588) | 0.193 | 0.027 | 0.209 |  |  |  |  |  |  |  |  |  |  |  |
| 5 (*C. chuyangsinensis* YHH 896) | 0.175 | 0.171 | 0.097 | 0.182 |  |  |  |  |  |  |  |  |  |  |
| 6 (*C. compactiuscula* YFCC 897) | 0.180 | 0.116 | 0.199 | 0.107 | 0.172 |  |  |  |  |  |  |  |  |  |
| 7 (*C. kunmingensis* YFCC 898) | 0.161 | 0.020 | 0.204 | 0.042 | 0.182 | 0.121 |  |  |  |  |  |  |  |  |
| 8 (*C. pseudochroleuca* CBS 187.94) | 0.172 | 0.024 | 0.222 | 0.031 | 0.188 | 0.121 | 0.024 |  |  |  |  |  |  |  |
| 9 (*C. rhizophaga* YFCC 900) | 0.187 | 0.027 | 0.204 | 0.045 | 0.171 | 0.126 | 0.023 | 0.042 |  |  |  |  |  |  |
| 10 (*C. rogersoniana* YFCC 899) | 0.183 | 0.053 | 0.182 | 0.068 | 0.173 | 0.121 | 0.065 | 0.073 | 0.077 |  |  |  |  |  |
| 11 (*C. rosea* YFCC 893) | 0.201 | 0.027 | 0.228 | 0.027 | 0.194 | 0.117 | 0.042 | 0.031 | 0.053 | 0.078 |  |  |  |  |
| 12 (*C. solani* YFCC 901) | 0.195 | 0.017 | 0.216 | 0.031 | 0.171 | 0.112 | 0.031 | 0.027 | 0.042 | 0.057 | 0.031 |  |  |  |
| 13 (*C. viticola* MUM 18.51) | 0.182 | 0.030 | 0.208 | 0.037 | 0.181 | 0.106 | 0.041 | 0.034 | 0.052 | 0.068 | 0.038 | 0.027 |  |  |
| 14 (*C. wenpingii* HMAS 172156) | 0.178 | 0.020 | 0.208 | 0.027 | 0.171 | 0.125 | 0.034 | 0.031 | 0.045 | 0.068 | 0.034 | 0.023 | 0.037 |  |

**TABLE S3** Pairwise genetic distance matrix of *Clonostachys* species for partial *TEF1* sequences

| Taxon | 1 | 2 | 3 | 4 | 5 | 6 | 7 | 8 | 9 | 10 | 11 | 12 | 13 | 14 |
| --- | --- | --- | --- | --- | --- | --- | --- | --- | --- | --- | --- | --- | --- | --- |
| 1 (*C. buxi* CBS 696.93) |  |  |  |  |  |  |  |  |  |  |  |  |  |  |
| 2 (*C. byssicola* CBS 364.78) | 0.333 |  |  |  |  |  |  |  |  |  |  |  |  |  |
| 3 (*C. candelabrum* CBS 504.67) | 0.328 | 0.315 |  |  |  |  |  |  |  |  |  |  |  |  |
| 4 (*C. chloroleuca* CBS 141588) | 0.336 | 0.026 | 0.288 |  |  |  |  |  |  |  |  |  |  |  |
| 5 (*C. chuyangsinensis* YHH 896) | 0.327 | 0.286 | 0.083 | 0.273 |  |  |  |  |  |  |  |  |  |  |
| 6 (*C. compactiuscula* YFCC 897) | 0.349 | 0.185 | 0.337 | 0.196 | 0.311 |  |  |  |  |  |  |  |  |  |
| 7 (*C. kunmingensis* YFCC 898) | 0.337 | 0.043 | 0.289 | 0.036 | 0.267 | 0.206 |  |  |  |  |  |  |  |  |
| 8 (*C. pseudochroleuca* CBS 187.94) | 0.348 | 0.068 | 0.287 | 0.061 | 0.253 | 0.186 | 0.080 |  |  |  |  |  |  |  |
| 9 (*C. rhizophaga* YFCC 900) | 0.314 | 0.040 | 0.308 | 0.040 | 0.279 | 0.186 | 0.058 | 0.061 |  |  |  |  |  |  |
| 10 (*C. rogersoniana* YFCC 899) | 0.354 | 0.132 | 0.281 | 0.143 | 0.266 | 0.191 | 0.156 | 0.130 | 0.147 |  |  |  |  |  |
| 11 (*C. rosea* YFCC 893) | 0.314 | 0.029 | 0.315 | 0.029 | 0.292 | 0.186 | 0.040 | 0.057 | 0.029 | 0.129 |  |  |  |  |
| 12 (*C. solani* YFCC 901) | 0.361 | 0.075 | 0.325 | 0.076 | 0.289 | 0.182 | 0.092 | 0.046 | 0.068 | 0.141 | 0.064 |  |  |  |
| 13 (*C. viticola* MUM 18.51) | 0.319 | 0.064 | 0.320 | 0.072 | 0.304 | 0.181 | 0.092 | 0.043 | 0.068 | 0.121 | 0.053 | 0.046 |  |  |
| 14 (*C. wenpingii* HMAS 172156) | 0.326 | 0.029 | 0.321 | 0.029 | 0.292 | 0.185 | 0.047 | 0.072 | 0.043 | 0.132 | 0.033 | 0.079 | 0.068 |  |
