# Supplementary material for: Phylogeny and systematics of the genus Clonostachys
Source: Front Microbiol. 2023 Mar 3;14:1117753. doi: 10.3389/fmicb.2023.1117753 (PMC10020229; doi:10.3389/fmicb.2023.1117753)
Supplement: Supplementary file 2 [file Data_Sheet_2.docx]

**
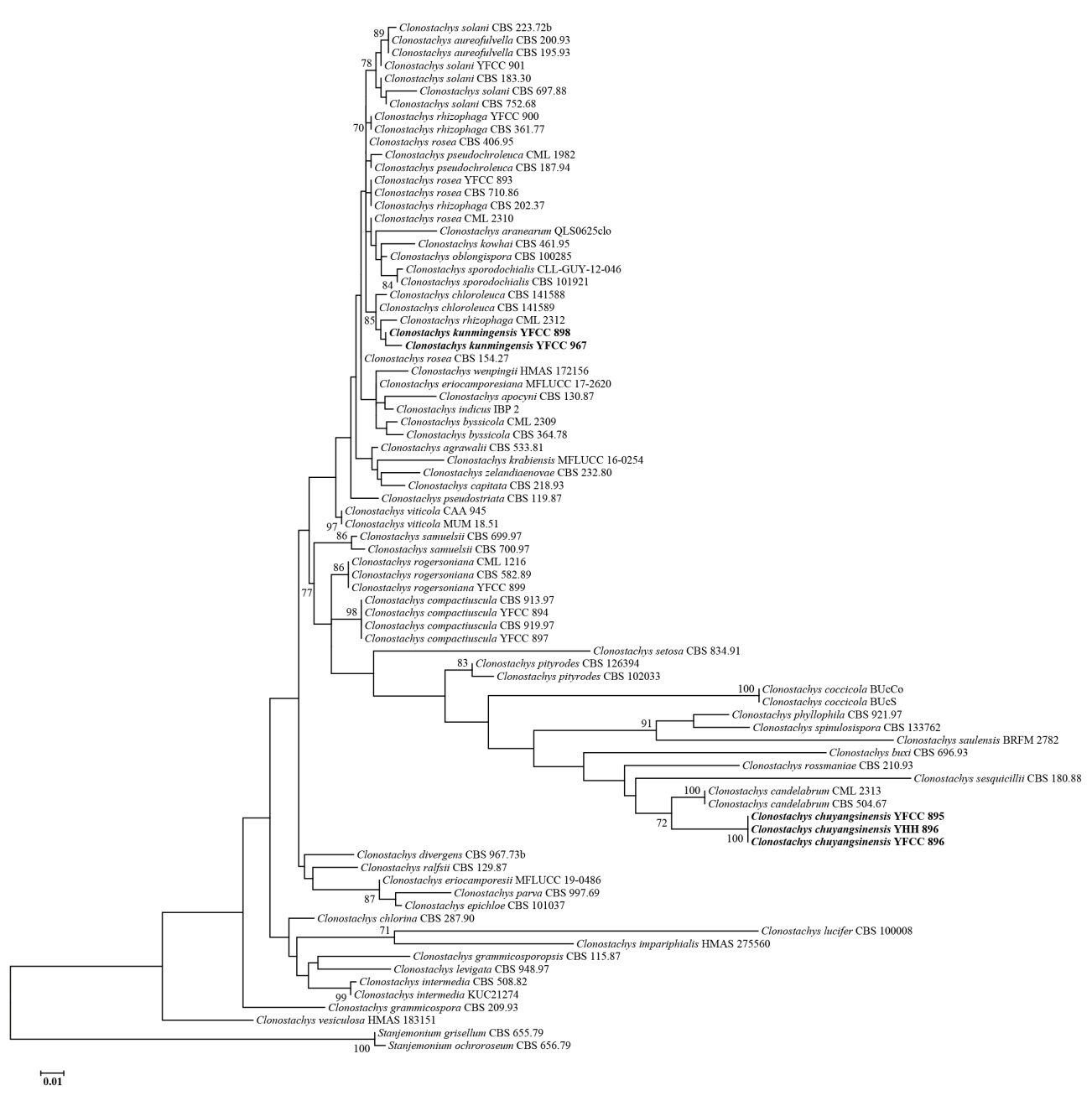
**

**FIGURE S1** Phylogenetic tree of *Clonostachys* based on Maximum Likelihood (ML) analysis from the ITS sequences. Statistical support values (≥70%) are shown at the nodes for ML boostrap support.


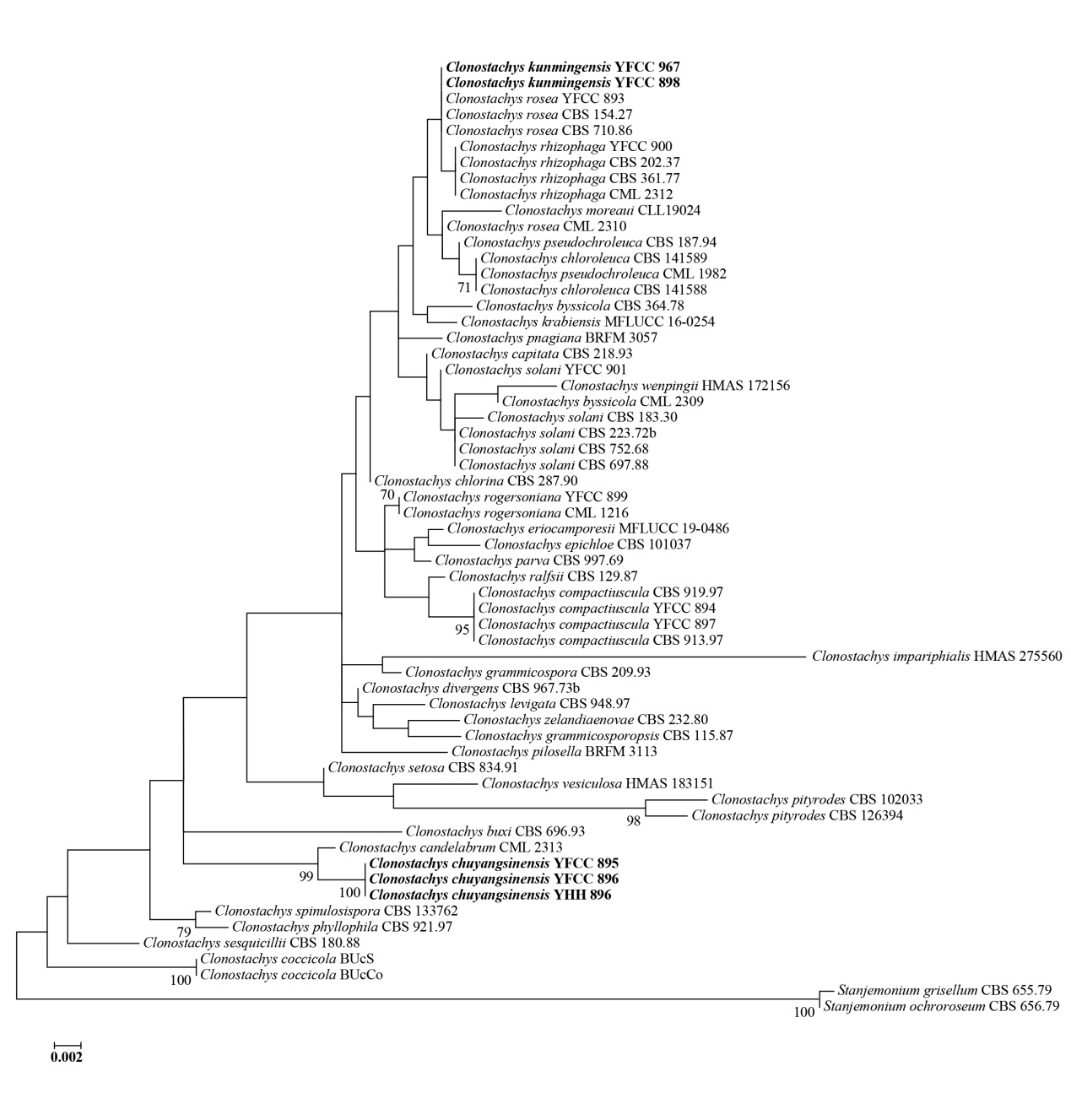


**FIGURE S2** Phylogenetic tree of *Clonostachys* based on Maximum Likelihood (ML) analysis from the nr*LSU* sequences. Statistical support values (≥70%) are shown at the nodes for ML boostrap support.


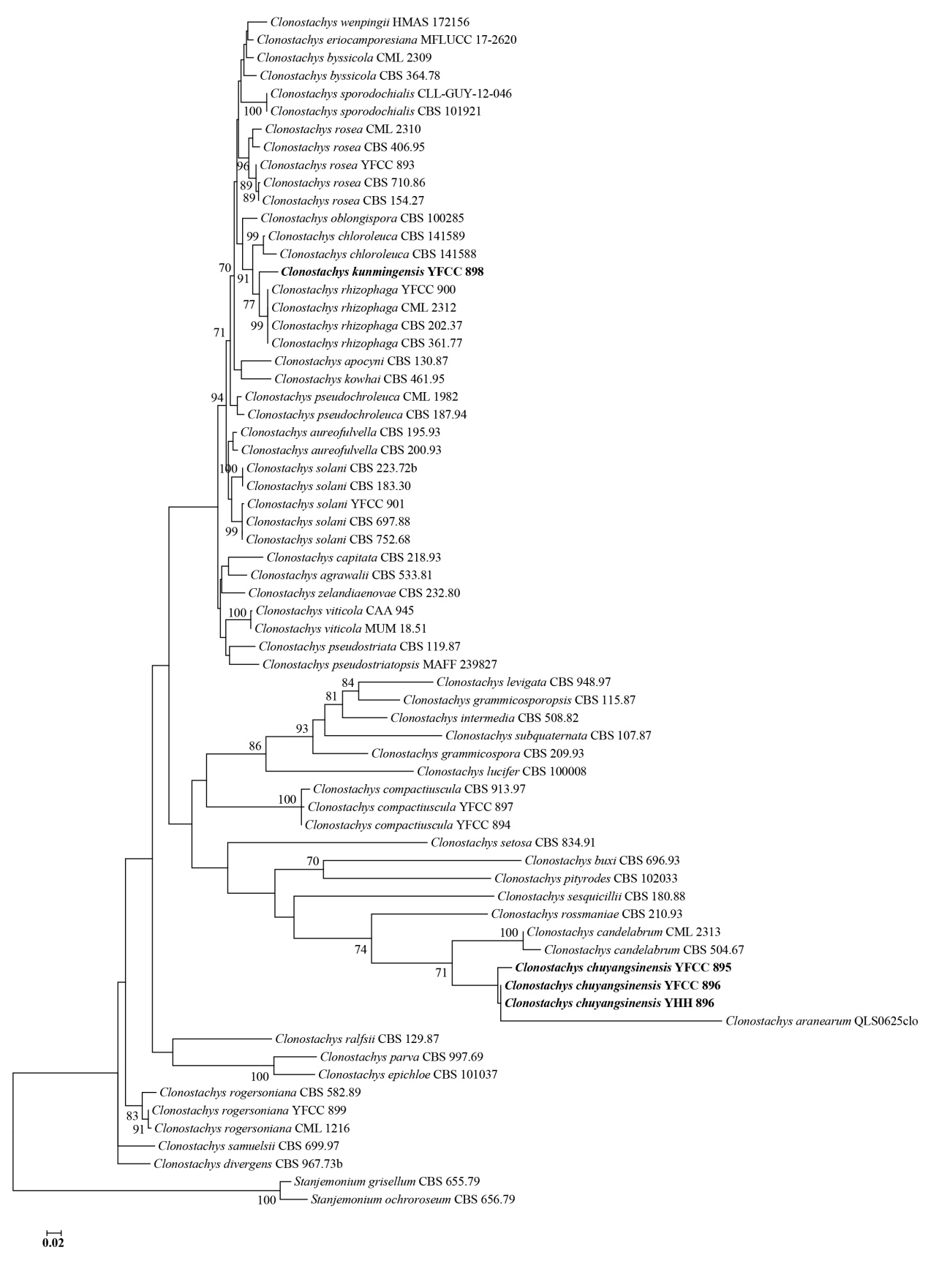


**FIGURE S3** Phylogenetic tree of *Clonostachys* based on Maximum Likelihood (ML) analysis from the *TUB2* sequences. Statistical support values (≥70%) are shown at the nodes for ML boostrap support.


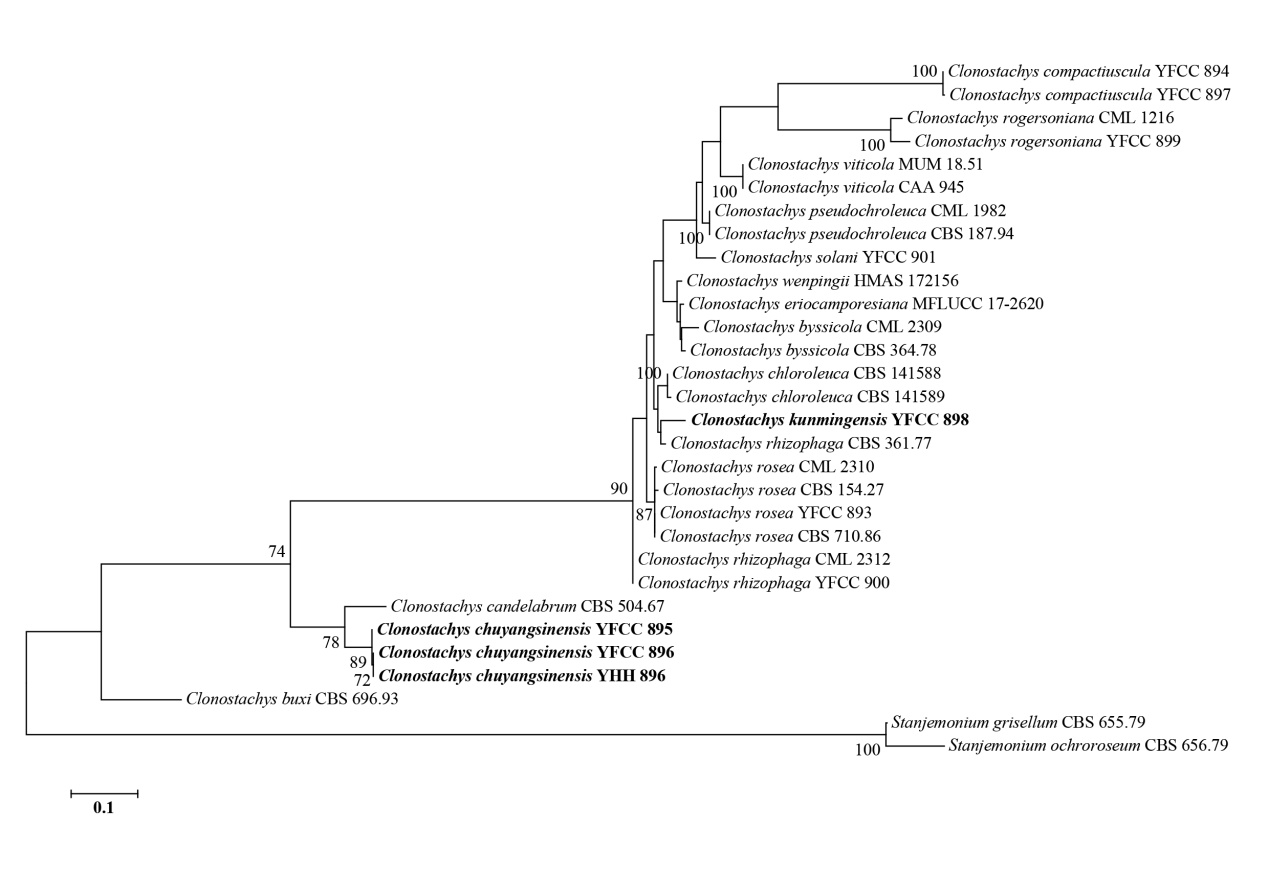


**FIGURE S4** Phylogenetic tree of *Clonostachys* based on Maximum Likelihood (ML) analysis from the *TEF1* sequences. Statistical support values (≥70%) are shown at the nodes for ML boostrap support.
